# The GLP‐1–Mitochondria Axis in Metabolic Aging

**DOI:** 10.1111/acel.70676

**Published:** 2026-08-17

**Authors:** Renin Chang, Andy P. Tsai, Boyang Wang, Chia‐Jung Li

**Affiliations:** ^1^ Department of Medical Education and Research Kaohsiung Veterans General Hospital Kaohsiung Taiwan; ^2^ Center of General Education, Cheng Shiu University Kaohsiung Taiwan; ^3^ Center of General Education, Shu‐Zen Junior College of Medicine and Management Kaohsiung Taiwan; ^4^ Department of Neurology and Neurological Sciences Stanford University School of Medicine Stanford California USA; ^5^ Sundial Initiative Palo Alto California USA; ^6^ Department of Obstetrics and Gynecology Kaohsiung Veterans General Hospital Kaohsiung Taiwan; ^7^ Institute of Biopharmaceutical Sciences, National Sun Yat‐Sen University Kaohsiung Taiwan

**Keywords:** geroscience, GLP‐1 receptor agonists, healthy aging, metabolic aging, mitochondrial dysfunction, mitochondrial resilience, population health

## Abstract

Metabolic aging underlies a cluster of chronic conditions—type 2 diabetes, cardiovascular disease, sarcopenia, and neurodegeneration—that account for a substantial share of global morbidity and mortality. A common feature is progressive mitochondrial dysfunction: impaired bioenergetics, disrupted quality control, and loss of metabolic resilience. Reduced mitochondrial DNA copy number in peripheral blood leukocytes is associated with cardiometabolic disease and mortality, but pre‐analytical variability, dependence on blood‐cell composition, and uncertain relationship to tissue‐level function mean it should be regarded as a candidate risk‐associated biomarker rather than a validated measure of mitochondrial integrity. Glucagon‐like peptide‐1 receptor agonists (GLP‐1 RAs), developed for glycemic control, engage pathways implicated in mitochondrial biogenesis, dynamics, and mitophagy; whether these effects reflect direct receptor signaling, indirect consequences of weight loss, or secondary mediators such as interleukin‐6 remains debated and appears tissue‐dependent. In SELECT, semaglutide reduced major adverse cardiovascular events by 20% in obesity without diabetes, and a 2025 multi‐omic study in aged male mice found GLP‐1 RA treatment attenuated age‐associated molecular signatures despite only modest changes in food intake and body weight. No trial, however, has incorporated a prespecified mitochondrial endpoint, human mechanistic evidence remains limited, and access to these therapies remains uneven worldwide. Here we synthesize mechanistic, preclinical, and clinical evidence for a proposed GLP‐1–mitochondria axis, classify this evidence by receptor dependence and translational stage, distinguish disease‐specific treatment effects from evidence for aging modification, examine four major controversies, and outline a research and policy agenda for responsible, evidence‐graded development of GLP‐1‐based geroscience interventions.

## Graphical abstract

Glucagon‐like peptide‐1 receptor agonists engage AMPK–PGC‐1α signaling to remodel mitochondrial biogenesis, dynamics, and quality control, but downstream effects are context‐dependent and tissue‐specific rather than uniformly receptor‐proximal. Whether this proposed mitochondria‐incretin axis reflects a novel geroscience mechanism or indirect metabolic effects remains an open, evidence‐graded question.

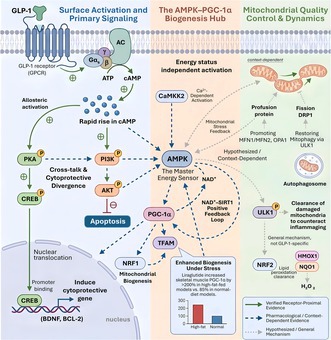

## 1 Introduction

The global aging transition represents one of the most consequential demographic shifts in human history. By 2050, the number of individuals aged 65 years and older is projected to exceed 1.6 billion, with most of this growth occurring in low‐ and middle‐income countries (Beard et al. 2016). As life expectancy increases, the major challenge facing healthcare systems is no longer simply extending lifespan, but preserving healthspan. Age‐related metabolic dysfunction underlies a cluster of highly prevalent non‐communicable diseases (NCDs), including type 2 diabetes mellitus (T2DM), cardiovascular disease (CVD), metabolic dysfunction–associated steatotic liver disease (MASLD), sarcopenia, and neurodegenerative disorders. Together, these conditions account for a substantial proportion of global morbidity, mortality, and healthcare expenditure (GBD 2019 Risk Factors Collaborators 2020). Identifying biological pathways that drive multiple age‐associated diseases simultaneously, while remaining amenable to scalable intervention, has therefore become a central goal of both geroscience and public health.

### 1.1 Definitions and Scope: Metabolic Aging, Healthspan, and Geroscience Criteria

Throughout this review we use “metabolic aging” to describe the age‐associated decline in mitochondrial and metabolic resilience that predisposes to, but is not synonymous with, individual diseases such as T2DM, CVD, MASLD, sarcopenia, and neurodegeneration. We distinguish this from several related but non‐interchangeable constructs: chronological age (time since birth); biological age (the cumulative burden of molecular and physiological damage, which may diverge from chronological age); healthspan (the period of life free from major disability or chronic disease); and lifespan (total survival). Clinical improvement in an individual age‐associated disease—for example, a reduction in major adverse cardiovascular events or histological improvement in MASH—demonstrates disease‐specific efficacy but does not, by itself, demonstrate modification of biological aging. Under a geroscience framework (Kennedy et al. 2014; Sierra and Kohanski 2017), an intervention would need to show effects on hallmark aging outcomes—frailty indices, physical performance, multimorbidity, disability‐free survival, cognitive function, resilience to physiological stress, or validated biological‐age measures—ideally across multiple organ systems and, ultimately, on lifespan, to be classified as geroprotective rather than disease‐specific. We use this distinction throughout to separate evidence that GLP‐1 RAs treat individual age‐related diseases from the substantially thinner evidence that they modify aging biology itself.

### 1.2 Search Strategy and Scope of This Review

This is a narrative, evidence‐informed review rather than a systematic review. Relevant literature was identified through structured searches of PubMed/MEDLINE and Google Scholar (last search: April 2026) using combinations of the terms “GLP‐1”, “glucagon‐like peptide‐1”, “incretin”, “mitochondria”, “mitochondrial biogenesis”, “AMPK”, “PGC‐1α”, “mitophagy”, “mtDNA copy number”, “geroscience”, “sarcopenia”, and “aging”, supplemented by hand‐searching reference lists and citing articles of key papers. No date restriction was applied to foundational mechanistic literature underlying this review (AMPK signaling, mitochondrial dynamics, and NAD+‐sirtuin biology established prior to their application to GLP‐1 pharmacology); GLP‐1‐specific clinical, preclinical, and population‐level literature was prioritized from 2016 onward, reflecting the period during which cardiovascular outcome trials and mechanistic studies in this area became available, with particular emphasis on studies published in 2023–2026. English‐language mechanistic, preclinical, and clinical studies were prioritized, with additional weight given to randomized trials and systematic reviews/meta‐analyses. Because this is a narrative rather than systematic review, study selection is not exhaustive and is subject to selection bias; where possible we prioritized comprehensive systematic reviews and meta‐analyses (Karakasis et al. 2025; Old et al. 2025) over individual primary studies to reduce this risk. This review's intended contribution relative to prior overviews of GLP‐1 receptor agonists and aging‐related pathology (Peng et al. 2022; Wilbon and Kolonin 2023), healthy‐lifespan extension (Chavda et al. 2024; Kreiner et al. 2023), and skeletal‐muscle mitochondrial function specifically (Old et al. 2025) is to integrate mechanistic, preclinical, and clinical evidence into an explicitly evidence‐graded framework that separates receptor‐proximal, pharmacological, and indirect mechanisms, and to connect this framework to population‐scale biomarker and outcomes data and to a formal geroscience‐criteria discussion (Section 1.1). The mechanistic body of this review concerns GLP‐1 receptor agonism specifically; dual GLP‐1/GIP and triple GLP‐1/GIP/glucagon agonists are discussed only insofar as clinical and body‐composition data exist, and direct GIP‐receptor‐mediated mitochondrial mechanisms are not established and are not claimed here (see Section 6.4 for further discussion of receptor specificity across agonist classes). We use “GLP‐1” rather than “incretin” throughout, including in the title, to keep the framework's name aligned with the receptor system for which mechanistic mitochondrial evidence actually exists.

Mitochondria are increasingly recognized as a central integrator of metabolic aging, linking nutrient sensing, cellular energetics, inflammatory signaling, and tissue resilience. Age‐associated deterioration of mitochondrial function contributes to impaired metabolic flexibility, chronic low‐grade inflammation, and loss of cellular homeostasis. Importantly, mitochondrial dysfunction is not merely a cellular phenomenon. Large epidemiological studies demonstrate that reduced mitochondrial DNA (mtDNA) copy number in peripheral blood leukocytes independently predicts incident T2DM, cardiovascular disease, and all‐cause mortality, which supports mtDNA‐CN as a candidate risk‐associated biomarker of population‐level metabolic health, with the caveats on analytical and biological interpretation discussed in Section 2.3 and Table 1 (Fu et al. 2025; Huang et al. 2023; Lopez‐Otin et al. 2023; Malyutina et al. 2023; Sundquist et al. 2022).

**TABLE 1:** Candidate mitochondrial biomarkers of metabolic aging.

Biomarker	What it measures	Analytical validity	Biological interpretation	Current context of use
mtDNA‐CN, peripheral blood leukocytes	Relative abundance of mitochondrial genome copies per cell	Assay‐platform dependent (qPCR vs. sequencing); confounded by leukocyte subtype mix and platelet contamination; substantial pre‐analytical variation	Association with metabolic/CV outcomes and mortality is well replicated at the population level; relationship to tissue‐level respiratory function is not established	Candidate risk‐associated biomarker; not a validated surrogate for mitochondrial function or treatment response
Circulating NAD+ metabolites	Systemic NAD+ precursor/metabolite pool	Multiple assay methods, limited cross‐study standardization	Reflects a pharmacodynamic cofactor pool relevant to sirtuin activity, not a direct mitochondrial‐function readout	Investigational; trials of NAD+ precursors have produced inconsistent functional benefit
Cell‐free mitochondrial DNA	mtDNA fragments released into circulation	Sensitive to sample handling/hemolysis; no consensus reference method	Reflects tissue damage/mtDNA release and innate‐immune (cGAS‐STING) activation rather than organellar abundance	Investigational inflammatory/damage marker; not interchangeable with mtDNA‐CN

At the same time, glucagon‐like peptide‐1 receptor agonists (GLP‐1 RAs) have emerged as one of the most transformative therapeutic classes in metabolic medicine. Originally developed for glycemic control, GLP‐1 RAs have demonstrated benefits extending beyond glucose lowering, including reductions in cardiovascular risk, body weight, and obesity‐related complications. In the SELECT trial, semaglutide reduced major adverse cardiovascular events by 20% among individuals with obesity without diabetes (Lincoff et al. 2023). More recently, a landmark multi‐omic study reported that GLP‐1 RA treatment partially reversed multiple age‐associated molecular signatures across tissues in aged male mice, with sex‐specific responses not assessed and no lifespan or validated healthspan endpoint examined (Section 4.1) (Huang et al. 2025). Together, these findings have raised the possibility that incretin‐based therapies may influence fundamental mechanisms of aging rather than simply treating individual diseases. We propose, as a hypothesis to be evaluated against the evidence graded in Table 2 rather than as an established finding, that the GLP‐1–mitochondria axis provides a unifying framework through which incretin signaling coordinates mitochondrial quality control, bioenergetic adaptation, and stress resilience across multiple organ systems, thereby linking metabolic regulation to healthy aging.

**TABLE 2:** Evidence classification for proposed GLP‐1–mitochondria mechanisms.

Proposed effect	Tissue/model	Agent(s) tested	Receptor dependence tested?	Evidence tier
AMPK‐PGC‐1α mitochondrial biogenesis	Skeletal muscle, high‐fat‐fed mice (review‐level synthesis)	Multiple GLP‐1 RAs	Not tested with antagonist/KO	Pharmacological
Adipose browning/thermogenesis via IL‐6‐STAT3	Adipose tissue, mouse; monocytes, human	Liraglutide, exenatide	Yes—adipose IL‐6R knockout abolishes effect; GLP‐1R not required	Indirect/secondary
Mitochondrial uncoupling, SIRT1/3 induction	Human adipocytes (Chub‐S7, ex vivo)	Exendin‐4	Yes—abolished by GLP‐1R antagonist	Receptor‐proximal
Leukocyte mitochondrial respiration and redox state	Peripheral blood leukocytes, T2DM patients	Mixed GLP‐1 RAs	Not tested	Clinical, observational
Skeletal‐muscle mitochondrial response, healthy vs. lipotoxic	C2C12 myotubes, in vitro	Semaglutide, tirzepatide, cagrilintide	Not tested	Pharmacological, cell‐based
Cardiac GLP‐1R protein expression	Human heart (4 chambers, SA node)	N/A (receptor mapping)	N/A	Receptor mapping
Body‐wide multi‐omic aging reversal	Multi‐tissue, aged male mice only	GLP‐1 RA (agent as reported)	Not tested	Preclinical, systems‐level
Lean‐mass change with weight loss	22 RCTs, network meta‐analysis	GLP‐1 RAs and GLP‐1/GIP co‐agonists	N/A	Clinical, meta‐analysis
Relative muscle mass/strength, running performance	Obese mice + human proof‐of‐concept	GLP‐1 medicines (agents as reported)	Not tested	Preclinical + limited clinical
MACE reduction	LEADER, SUSTAIN‐6, SELECT, SURPASS‐CVOT	Liraglutide, semaglutide, tirzepatide	Not tested; no mitochondrial endpoint	Clinical RCT
Atrial fibrillation risk reduction	Meta‐analysis of RCTs, overweight/obesity	GLP‐1 RAs and co‐agonists	Not tested; no weight‐loss‐magnitude interaction	Clinical, meta‐analysis

Despite this promise, important translational challenges remain. Much of the mechanistic evidence linking GLP‐1 receptor signaling to mitochondrial remodeling derives from cell and animal studies, whereas large clinical trials have yet to incorporate standardized mitochondrial functional endpoints. Furthermore, although tens of millions of individuals worldwide are now treated with GLP‐1‐based therapies, access remains highly uneven across populations and healthcare systems, raising important questions regarding affordability, equity, and sustainable implementation. Whether mitochondrial remodeling directly mediates the broad clinical benefits of GLP‐1 RAs, as opposed to reflecting indirect consequences of weight loss and improved metabolic homeostasis, is a central open question addressed systematically in Section 3.1.

In this review, we synthesize mechanistic, preclinical, clinical, and population‐level evidence for the proposed GLP‐1–mitochondria axis. We evaluate the strengths and limitations of current evidence using the explicit tiered framework introduced in Section 3.1, discuss four major controversies in the field, and propose a research and policy agenda for the responsible development of GLP‐1‐based geroscience interventions.

## 2 Mitochondrial Dysfunction as a Central Hallmark of Metabolic Aging

Mitochondria are increasingly recognized as central integrators of metabolic aging, linking nutrient sensing, bioenergetics, inflammatory signaling, and cellular resilience. Reflecting this role, the 2023 update to the Hallmarks of Aging framework identified mitochondrial dysfunction as one of the core interconnected drivers of biological aging (Lopez‐Otin et al. 2023). Progressive deterioration of mitochondrial function contributes to impaired metabolic flexibility, chronic inflammation, and loss of tissue homeostasis across multiple age‐related diseases. Understanding the molecular mechanisms underlying mitochondrial decline is therefore essential for identifying therapeutic strategies capable of targeting metabolic aging at its source.

### 2.1 The NAD ^+^‐Sirtuin Axis and Bioenergetic Decline

A defining biochemical feature of metabolic aging is the progressive decline in intracellular NAD^+^ availability. Beyond its role as an essential cofactor for oxidative phosphorylation (OXPHOS), NAD^+^ serves as the principal substrate for the sirtuin family of deacetylases, particularly SIRT1 and the mitochondrial deacetylase SIRT3 (Amjad et al. 2021; Yusri et al. 2025). Age‐associated NAD+ depletion—driven in part by increased consumption by poly(ADP‐ribose) polymerases (PARPs) activated by cumulative DNA damage—impairs sirtuin activity, suppresses PGC‐1α‐dependent mitochondrial biogenesis, reduces electron transport chain efficiency, and weakens antioxidant defenses (Canto et al. 2009; Verdin 2015). These changes establish a self‐reinforcing cycle in which mitochondrial dysfunction promotes oxidative stress, further compromising mitochondrial quality and metabolic resilience. Given its central role in coordinating bioenergetics and stress responses, the NAD^+^‐sirtuin axis has emerged as a key therapeutic target in aging biology.

### 2.2 Mitochondrial Dynamics, Mitophagy, and Inflammaging

Mitochondrial function depends not only on energy production but also on the continuous maintenance of mitochondrial quality through coordinated fusion, fission, and mitophagy. Aging disrupts these processes, resulting in fragmentation of the mitochondrial network and accumulation of dysfunctional organelles (Westermann 2010). Concurrent impairment of mitophagy (coordinated by the PINK1–Parkin and AMPK–ULK1 pathways, which decline in aged skeletal muscle (Seabright and Lai 2020; Singh et al. 2024)) limits the efficient removal of damaged mitochondria, increasing reactive oxygen species production and the release of mitochondrial danger signals, including mitochondrial DNA fragments (Pickrell and Youle 2015). These signals activate innate immune pathways that contribute to chronic low‐grade inflammation, or inflammaging, thereby linking mitochondrial dysfunction to systemic metabolic decline and age‐related disease susceptibility (Decout et al. 2021); in particular, mislocalized mtDNA activates the cytosolic cGAS–STING pathway, which has been shown to drive aging‐related inflammation and neurodegeneration in vivo (Gulen et al. 2023). This mitochondrial‐dysfunction–senescence relationship is bidirectional and mechanistically deeper than a single downstream inflammatory readout: dysfunctional mitochondria can themselves promote entry into a senescent, secretory state, and senescent cells in turn reshape mitochondrial metabolism, so that mitochondrial decline and cellular senescence form a reinforcing pair of aging hallmarks rather than a strictly linear cascade. Whether GLP‐1 receptor signaling intersects proteostasis pathways further upstream (e.g., the mitochondrial unfolded protein response (UPRmt), which coordinates a transcriptional response to misfolded mitochondrial protein and has not been directly tested in the context of GLP‐1 RA treatment) is speculative and is listed as a research priority in Table 3 rather than presented as an established mechanism.

**TABLE 3:** Priority research questions for the GLP‐1–mitochondria field.

#	Research question	Preferred design	Comparison groups required [NEW]	Feasible mitochondrial endpoints [NEW]
1	Are GLP‐1 RA mitochondrial benefits direct or indirect?	Tissue‐specific GLP‐1R knockout + organ‐level metabolomics	GLP‐1R‐KO/antagonist arm; wild‐type + vehicle arm	Mitochondrial proteomics; tissue‐specific metabolic flux
2	Do therapeutic doses restore skeletal muscle mitochondrial respiratory capacity in humans?	RCT with pre‐specified mtDNA‐CN and ex vivo respirometry	Placebo‐controlled; pair‐weight‐matched caloric‐restriction arm	High‐resolution respirometry; 31P‐MRS phosphocreatine recovery
3	Does resistance exercise + GLP‐1 RA preserve mitochondrial quality and lean mass in older adults (≥ 60 y, incl. nonobese safety data, Section 6.5)?	2 × 2 factorial RCT, adults aged ≥ 60	GLP‐1 RA alone; exercise alone; combination; placebo/no exercise	mtDNA integrity; mitophagy reporters; grip strength/gait speed
4	Do dual/triple agonists (tirzepatide, retatrutide) provide greater or distinct mitochondrial benefits, and is any GIP‐receptor‐specific effect separable from the shared GLP‐1 component?	Head‐to‐head RCT with standardized mitochondrial biomarker panel	Mono‐ vs. dual‐ vs. triple‐agonist arms; GIPR‐selective comparator if available	Mitochondrial proteomics; mtDNA‐CN; respirometry
5	Do mtDNA‐CN and NAD+ metabolomics predict GLP‐1 RA mitochondrial response, and does target engagement mediate clinical outcome?	Nested sub‐study in CVOTs; Mendelian randomization; formal mediation analysis	Treated vs. untreated within trial; genetically‐proxied GLP‐1R activity	Longitudinal mtDNA‐CN; NAD+ metabolomics; cf‐mtDNA

*Note:* Addressing these priorities will require closer integration of geroscience, cardiometabolic medicine, and population health research. Future trials should be designed not only to evaluate clinical efficacy, but also to clarify mechanisms, validate mitochondrial biomarkers, and identify the populations most likely to benefit. Generating this evidence will be essential for determining whether the promise of the GLP‐1–mitochondria axis can be translated into equitable and scalable strategies for promoting healthy aging.

### 2.3 From Organelle to Population: Mitochondrial Biomarkers of Metabolic Health

**FIGURE 1:**
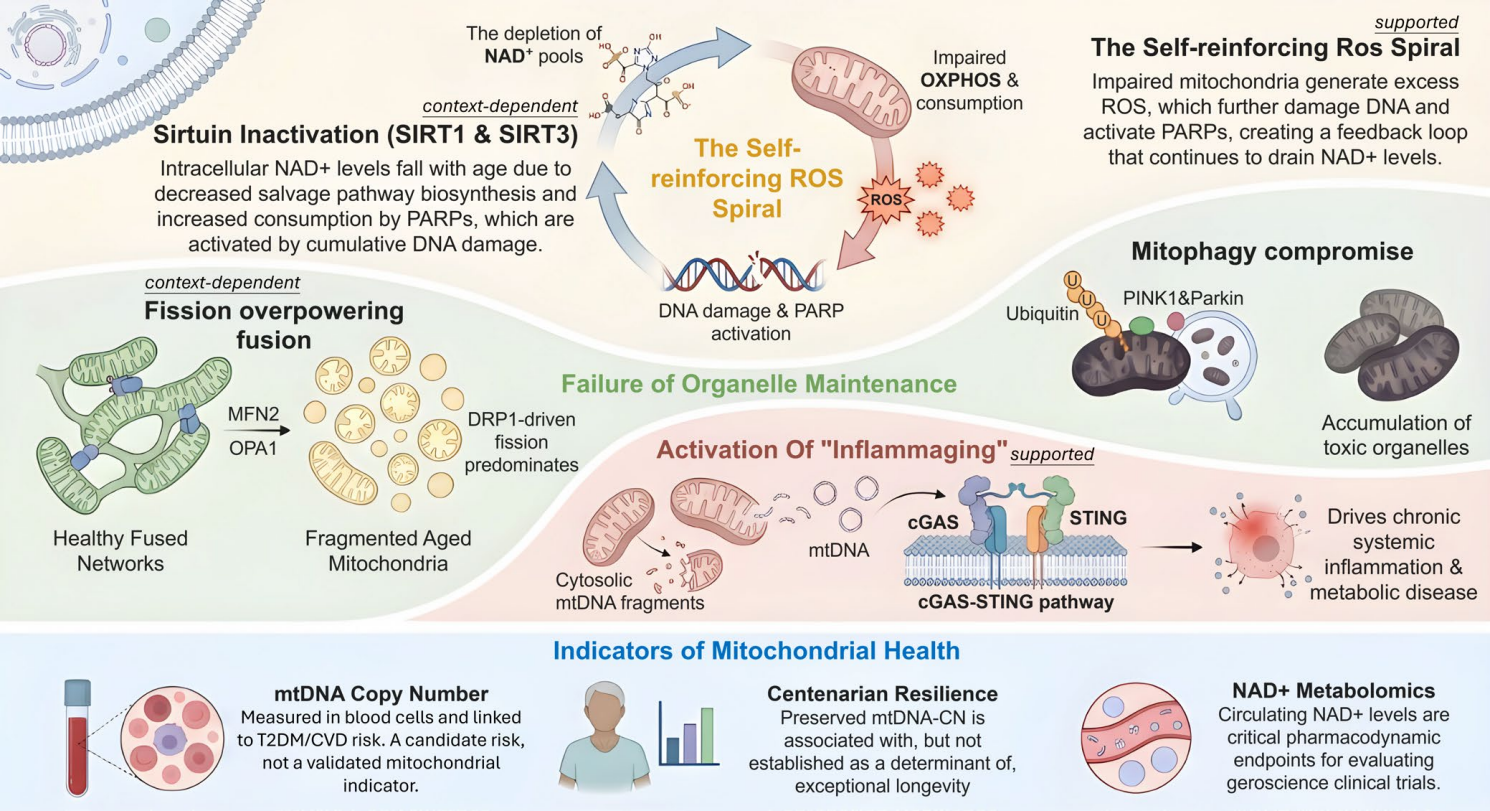
The self‐reinforcing cycle of mitochondrial dysfunction in metabolic aging. Age‐associated decline in NAD+ availability impairs sirtuin‐dependent mitochondrial quality control, suppresses PGC‐1α‐mediated mitochondrial biogenesis, and reduces oxidative phosphorylation (OXPHOS) efficiency. The resulting increase in reactive oxygen species (ROS) further damages mitochondrial function, reinforcing a cycle of bioenergetic decline. Concurrent disruption of mitochondrial dynamics and mitophagy promotes the accumulation of dysfunctional mitochondria and amplifies pro‐inflammatory signaling, contributing to chronic low‐grade inflammation (inflammaging). At the population level, reduced mitochondrial DNA copy number (mtDNA‐CN) in peripheral blood leukocytes is associated with increased risk of type 2 diabetes, cardiovascular disease, and all‐cause mortality; mtDNA‐CN is a candidate risk‐associated biomarker and not a validated measure of tissue mitochondrial integrity (Section 2.3, Table 1).

The recognition of mitochondrial dysfunction as a driver of metabolic aging has stimulated interest in scalable biomarkers of mitochondrial health. Among these, mitochondrial DNA copy number (mtDNA‐CN) in peripheral blood leukocytes is the most extensively studied. Large prospective cohort studies demonstrate that lower mtDNA‐CN is associated with incident T2DM, cardiovascular disease, and all‐cause mortality, even after adjustment for conventional risk factors (Fu et al. 2025; Huang et al. 2023; Li et al. 2024; Malyutina et al. 2023; Sundquist et al. 2022); Conversely, preservation of mtDNA‐CN has been associated with exceptional longevity (Garagnani et al. 2014). However, mtDNA‐CN measured in peripheral blood is subject to substantial pre‐analytical variation, is strongly influenced by leukocyte subtype composition and platelet contamination, varies by assay platform (qPCR versus sequencing‐based methods are not directly comparable), and its relationship to mitochondrial respiratory function in skeletal muscle, liver, heart, or brain is not established. Its responsiveness to therapeutic intervention is also uncertain. For these reasons, mtDNA‐CN should currently be regarded as a candidate risk‐associated biomarker rather than a validated surrogate endpoint for tissue‐level mitochondrial resilience (Table 1). Emerging biomarkers, including circulating NAD+ metabolites and cell‐free mitochondrial DNA, measure different biological phenomena from mtDNA‐CN and from each other—NAD+ metabolites reflect a pharmacodynamic cofactor pool, cell‐free mtDNA reflects tissue damage/release, and mtDNA‐CN reflects organellar genome abundance—and should not be grouped together as an interchangeable “mitochondrial panel”; clinical trials of NAD+ precursors have to date produced inconsistent functional benefits (Prokopidis et al. 2025). Establishing standardized mitochondrial biomarker panels will be essential for integrating mitochondrial endpoints into future geroscience and population‐health studies. The interconnected mechanisms underlying mitochondrial aging are summarized in Figure 1 (Box 1).

BOX 1 **Tissue heterogeneity in mitochondrial aging.**

The manifestations of mitochondrial aging vary substantially across tissues according to their metabolic demands and regenerative capacity. In skeletal muscle, declines in mitochondrial content and OXPHOS capacity contribute to reduced aerobic fitness and sarcopenia. In the liver, impaired mitochondrial fatty acid oxidation promotes lipid accumulation and the development of metabolic dysfunction–associated steatotic liver disease (MASLD). In the aging heart, loss of metabolic flexibility compromises energetic reserve and increases vulnerability to cardiovascular stress. Neurons are particularly susceptible to cumulative mitochondrial damage because of their exceptionally high energy requirements and limited regenerative capacity. Despite these tissue‐specific manifestations, impaired mitochondrial quality control and loss of metabolic resilience represent common features across organ systems. This heterogeneity highlights the importance of evaluating mitochondrial‐targeted interventions across multiple tissues and clinical outcomes rather than relying on a single organ‐specific endpoint.

## 3 The GLP‐1‐Mitochondria Axis: Mechanisms

### 3.1 Direct Receptor‐Mediated Versus Indirect Mechanisms: An Evidence Framework

A central and unresolved controversy is whether the mitochondrial effects attributed to GLP‐1 RAs reflect direct receptor signaling within the tissue examined, or are secondary to systemic changes (such as weight loss, improved glycemia, reduced lipotoxicity, and lower inflammation) that themselves improve mitochondrial function. We classify evidence throughout this review into three tiers: (i) receptor‐proximal effects demonstrated by GLP‐1 receptor antagonism or genetic deletion; (ii) pharmacological effects observed with a GLP‐1 RA but without direct proof of receptor dependence; and (iii) indirect or secondary effects, mediated by weight loss, glycemic improvement, or a non‐GLP‐1R mediator. Table 2 applies this classification to every major line of evidence discussed below.

By the antagonist/knockout standard, direct evidence for GLP‐1R‐mediated mitochondrial effects is strongest in tissues with well‐validated GLP‐1R protein expression (pancreatic islets and, more recently, subsets of atrial and ventricular cardiomyocytes and the sinoatrial node) and considerably weaker elsewhere. Even cardiac GLP‐1R protein localization required careful, antibody‐validated mapping across multiple human tissue biobanks, and definitive ventricular cardiomyocyte protein localization remains incompletely resolved despite detectable mRNA transcripts (Baggio et al. 2018). GLP‐1R protein expression in human hepatocytes, skeletal myocytes, and adipocytes remains contested; several studies have failed to detect robust receptor protein in these tissues despite clear metabolic responses to GLP‐1 RA treatment, and validated antisera have historically lacked sensitivity for reliable immunodetection (Baggio et al. 2018; Wilbon and Kolonin 2023). This tissue‐expression uncertainty has motivated interest in indirect, receptor‐independent mediators of GLP‐1 RA action. One well‐characterized candidate is interleukin‐6 (IL‐6): GLP‐1 analogues acutely induce monocyte IL‐6 secretion in prediabetic humans, and in mice, GLP‐1 analogue‐induced IL‐6/IL‐6 receptor signaling in adipose tissue (rather than direct adipocyte GLP‐1R engagement) drives STAT3 activation, adipose browning, and thermogenesis; adipose‐specific IL‐6 receptor knockout abolishes the browning and thermogenic response while liraglutide‐induced weight loss is preserved, formally dissociating this receptor‐independent mitochondrial/thermogenic effect from the metabolic (weight‐loss) effect of the drug (Gutierrez et al. 2022). Because IL‐6 can activate AMPK in other tissue contexts, an IL‐6–AMPK relay is a plausible, receptor‐independent route by which circulating GLP‐1 RA‐induced IL‐6 could engage the AMPK‐PGC‐1α programme described in Section 3.2 within cells that express little or no GLP‐1R.

Mono‐ (semaglutide, liraglutide, dulaglutide, and exenatide), dual‐ (tirzepatide; GLP‐1/GIP), and triple‐hormone‐receptor agonists (retatrutide; GLP‐1/GIP/glucagon) are pharmacologically distinct, and mitochondrial and body‐composition data obtained with one class cannot be assumed to generalize to another; Table 2 records which specific agent was used for each line of evidence discussed below, and Section 6.4 discusses GIP‐receptor biology and receptor specificity further.

### 3.2 The AMPK‐PGC‐1α Axis and Mitochondrial Biogenesis

A defining feature of GLP‐1 RA action in the tissues where it has been studied is activation of AMP‐activated protein kinase (AMPK), a master regulator of cellular energy homeostasis (Canto and Auwerx 2010). AMPK stimulates the transcriptional coactivator PGC‐1α, which coordinates mitochondrial biogenesis through downstream activation of nuclear respiratory factors and mitochondrial transcription factor A (TFAM) (Scarpulla 2008). The net effect is expansion of mitochondrial capacity, improved oxidative phosphorylation, and enhanced ATP production. Notably, the magnitude of this response appears greatest in metabolically compromised tissues, suggesting that GLP‐1 RAs preferentially restore mitochondrial function under conditions of metabolic stress (Old et al. 2025). This cAMP→PKA/Epac→AMPK→PGC‐1α cascade is inferred largely from mono‐agonist (GLP‐1R) pharmacology. Whether dual and triple agonists engage the same cAMP‐centered signaling architecture, or instead activate GIP‐ and glucagon‐receptor‐specific programmes in parallel, has not been directly tested and is listed as an open research priority (Table 3); we are not aware of published preclinical data directly comparing mitochondrial or aging‐related effects of mono‐ versus dual/triple agonists.

### 3.3 The NAD ^+^ Signaling, Oxidative Stress and Mitochondrial Quality Control

**FIGURE 2:**
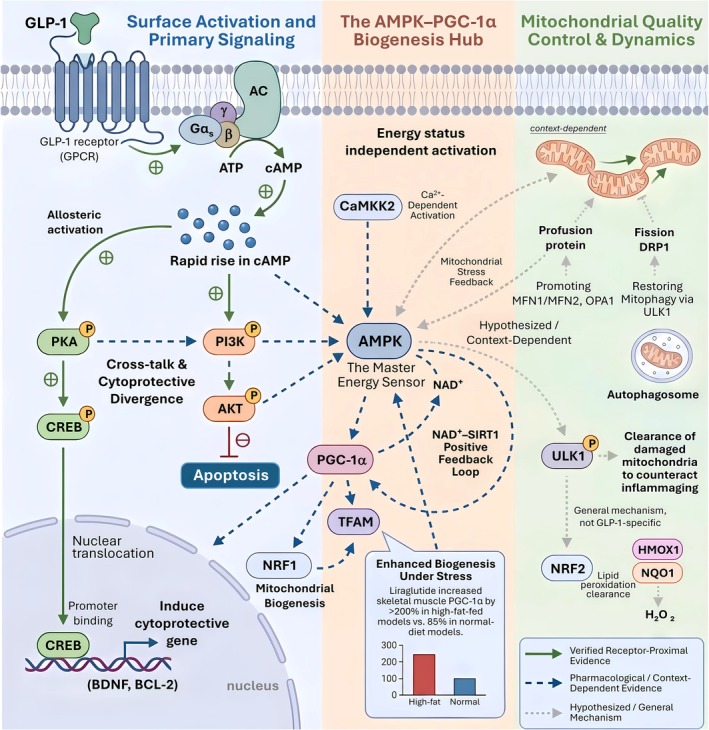
The GLP‐1–mitochondria signaling axis and tissue‐specific consequences of mitochondrial remodeling. GLP‐1 RA binding activates adenylyl cyclase, raising intracellular cAMP and engaging the PKA/CREB and PI3K/Akt (activating) pathways, which promote cytoprotective gene expression and suppress apoptosis. Downstream, CaMKK2‐mediated activation of AMPK initiates a mitochondrial remodeling programme centred on PGC‐1α, which induces NRF1/GABP and TFAM to drive mitochondrial biogenesis. Increased NAD^+^ availability activates SIRT1, further enhancing PGC‐1α activity through a positive‐feedback loop. AMPK's effect on mitochondrial dynamics is context‐dependent (Section 3.4) rather than uniformly fusion‐promoting, and enhances mitophagy through ULK1 in models where this has been directly tested. GLP‐1 RAs also activate NFE2L2 (Nrf2)‐dependent antioxidant pathways in some systems, increasing HMOX1 and NQO1 expression and reducing oxidative stress. Tissue‐specific outcomes are drawn from heterogeneous evidence tiers detailed in Table 2 and should not be read as uniformly receptor‐proximal. NRF1/GABP (mitochondrial biogenesis) and NFE2L2/Nrf2 (antioxidant response) share the historical name “Nrf2” but are distinct factors. Fusion and fission are both shown as hypothesized/general given the limited, model‐specific nature of current GLP‐1‐specific evidence for either direction (Section 3.4).

AMPK activation also enhances NAD+‐dependent signaling, including SIRT1‐mediated activation of PGC‐1α, a mechanism established in general AMPK‐SIRT1 biology (Canto et al. 2009) that has not been directly confirmed for most GLP‐1 RAs in vivo. Concurrently, in cell and rodent models, GLP‐1 RAs have been reported to reduce oxidative stress and attenuate mitochondrial reactive oxygen species production, for example through AMPK‐dependent mitophagy in exendin‐4‐treated vascular smooth muscle in the context of diabetes‐associated vascular calcification (Chen et al. 2024; Drucker 2018). The general antioxidant transcriptional response engaged downstream of AMPK/ULK1 signaling, including Nrf2 (NFE2L2)‐Keap1‐mediated induction of HMOX1 and NQO1 (Figure 2), is well characterized in non‐GLP‐1 contexts (Bellezza et al. 2018) but has been directly demonstrated for GLP‐1 RAs in only a limited number of cell and tissue systems, and this citation is offered here as general pathway background rather than GLP‐1‐specific evidence. Together, these mechanisms are proposed to improve mitochondrial quality and help interrupt the cycle of oxidative damage that characterizes metabolic aging. The evidence is not uniformly positive, however: in human Chub‐S7 adipocytes, exendin‐4 modestly decreased mitochondrial membrane potential while increasing uncoupled oxygen consumption and SIRT1/SIRT3 gene expression—an effect the original authors interpreted as beneficial mitochondrial uncoupling and increased energy expenditure rather than mitochondrial injury, and one that was abolished by GLP‐1 receptor antagonism, indicating genuine receptor dependence for this specific, directionally unexpected effect (Goralska et al. 2017). This finding illustrates that “improved mitochondrial function” is not a single, unambiguous readout: uncoupling, biogenesis, and respiratory efficiency can move in different directions depending on the endpoint measured, and reviews of this literature (including our own initial draft) should not implicitly assume concordance among them.

### 3.4 Mitochondrial Dynamics and Mitophagy

Whether GLP‐1 RAs shift the mitochondrial fusion‐fission balance toward fusion, as sometimes implied in schematic summaries of this pathway, is less well established than is often suggested by such diagrams. AMPK is well known to promote mitochondrial fission under acute energetic stress (Toyama et al. 2016) and to link energy sensing to mitophagy via ULK1 phosphorylation (Egan et al. 2011); these are foundational findings in general AMPK biology, not GLP‐1‐specific evidence. Direct, GLP‐1‐specific evidence for a shift in the fusion‐fission balance is more limited: in a pulmonary hypertension model, GLP‐1 agonism reduced right‐ventricular mitochondrial dysfunction in part through GSK3β‐Drp1‐dependent fission inhibition (Zhao et al. 2026), and exendin‐4 engaged AMPK‐dependent mitophagy in the context of diabetes‐associated vascular calcification (Chen et al. 2024). AMPK–PINK1/Parkin signaling is an established regulator of skeletal‐muscle mitophagy in general (Seabright and Lai 2020; Singh et al. 2024); whether GLP‐1 RAs engage this specific axis in human skeletal muscle in vivo has not been directly tested. These effects would be expected to promote the selective removal of dysfunctional mitochondria, improve network integrity, and reduce pro‐inflammatory signaling associated with mitochondrial damage where they occur, though the tissue and stressor conditions under which they occur remain incompletely mapped for GLP‐1 RAs specifically. The GLP‐1‐mitochondria signaling axis and its tissue‐specific therapeutic outcomes are summarized in Figure 2, now revised to encode the three‐tier evidence classification described in Section 3.1 (see figure legend and design note below) (Box 2).

BOX 2 **Key mechanistic open question.**

Do the mitochondrial benefits of GLP‐1 receptor agonists arise from direct GLP‐1 receptor signaling within peripheral tissues, or are they secondary consequences of improved metabolic homeostasis, including weight loss, glycemic control, and reduced lipotoxicity? As detailed in Section 3.1 and Table 2, current evidence supports contributions from both mechanisms in different tissues—for example, GLP‐1R‐antagonist‐reversible effects in human adipocytes versus an IL‐6‐receptor‐dependent, GLP‐1R‐independent effect in adipose browning—and their relative importance varies across tissues and disease states rather than following a single rule. Resolving this question will require tissue‐specific GLP‐1R knockout models, organ‐level multi‐omic profiling, and biomarker‐enriched clinical trials incorporating longitudinal mitochondrial endpoints. Clarifying the causal relationship between GLP‐1 signaling and mitochondrial remodeling is essential for determining whether these therapies can be extended beyond obesity and diabetes to broader aging populations.

## 4 Clinical Evidence: Bridging Mechanisms to Health Outcomes

Determining whether GLP‐1 RAs improve health outcomes through mitochondrial remodeling requires careful evaluation of evidence across preclinical and clinical studies. Although mechanistic studies consistently demonstrate effects on mitochondrial biogenesis, quality control, and bioenergetic function in specific tissue and stressor contexts (Table 2), direct evidence in humans remains limited. A major methodological challenge is the near‐complete absence of mitochondrial functional endpoints in large clinical trials. This section evaluates current evidence from multi‐omic aging studies, cardiovascular outcome trials, and musculoskeletal investigations, highlighting established findings, unresolved questions, and key opportunities for future biomarker‐informed research.

### 4.1 Multi‐Omic Evidence for Age‐Counteracting Effects

The most comprehensive preclinical evidence to date comes from a 2025 study published in *Cell Metabolism*, in which aged male mice only treated with a GLP‐1 RA from 11 months of age for 30 weeks exhibited broad, multi‐tissue attenuation of age‐associated molecular changes across transcriptomic, proteomic, metabolomic, and epigenomic datasets (Huang et al. 2025); sex‐specific responses were not assessed, and the study did not examine lifespan or a validated healthspan endpoint (Section 1.1). Notably, these effects were largely restricted to older animals, whereas young adults treated with the same regimen showed minimal molecular responses. Furthermore, the intervention produced only modest effects on food intake and body weight, which is not sufficient to establish that the observed molecular changes were independent of caloric restriction or weight loss, though it argues against these being the sole explanation. Among the pathways most consistently restored were those related to mitochondrial function, energy metabolism, and cellular stress responses, supporting a central role for mitochondrial remodeling in the biological response to GLP‐1 RA treatment in this specific model. Although these findings do not establish direct effects on lifespan or healthspan, they provide some of the strongest systems‐level evidence to date that incretin‐based therapies can modulate molecular processes associated with aging in aged male mice. Whether comparable multi‐omic restoration occurs in older humans receiving therapeutic doses remains an important unanswered question and a critical priority for future translational studies.

This distinction between molecular remodeling and demonstrated modification of the aging trajectory is not merely semantic: the EVOKE and EVOKE+ trials of oral semaglutide in early‐stage symptomatic Alzheimer's disease illustrate it directly, with biomarker‐level improvements failing to translate into slowed clinical progression (Cummings et al. 2026)—a cautionary precedent, referenced again in Section 5.1, against treating molecular remodeling as a proxy for a modified aging trajectory anywhere in this review.

### 4.2 Cardiovascular Outcome Trials: Evidence Beyond Glycemic Control

Cardiovascular outcome trials (CVOTs) provide the strongest clinical evidence that the benefits of GLP‐1 RAs extend beyond glucose lowering. In LEADER, liraglutide reduced three‐point major adverse cardiovascular events (MACE) by 13% among 9340 patients with T2DM and high cardiovascular risk (Marso, Daniels, et al. 2016). SUSTAIN‐6 demonstrated a 26% reduction in MACE with semaglutide among 3297 participants (Marso, Bain, et al. 2016). The SELECT trial extended these findings to individuals without diabetes, showing that semaglutide 2.4 mg reduced MACE by 20% among 17,604 participants with overweight or obesity and established CVD (Lincoff et al. 2023). The magnitude of cardiovascular benefit in these trials has motivated interest in mechanisms beyond glucose and weight reduction alone, though isolating any specific contribution of mitochondrial remodeling from these trial designs is not possible; these trials were not designed to test the GLP‐1–mitochondria framework and did not include tissue‐level mitochondrial measures and should be read as evidence of clinical benefit that motivates mechanistic investigation rather than as indirect validation of that framework (Table 2). For tirzepatide, the SURPASS‐CVOT trial demonstrated non‐inferiority to dulaglutide for MACE (HR 0.92) in T2DM with high cardiovascular risk (Nicholls et al. 2025).

Beyond atherosclerotic events, the STEP‐HFpEF programme showed that semaglutide improved symptoms, physical limitations, and exercise function and reduced heart‐failure events in obesity‐related heart failure with preserved ejection fraction, both without and with diabetes (Butler et al. 2024; Kosiborod et al. 2023; Kosiborod et al. 2024)—a phenotype closely tied to myocardial energetic impairment and reduced metabolic flexibility. In the liver, the Phase 3 ESSENCE trial demonstrated that semaglutide improved both steatohepatitis and fibrosis in patients with MASH (Sanyal et al. 2025), a result that is compatible with, but does not establish, the mitochondrial fatty‐acid‐oxidation mechanisms discussed in Section 3. None of these trials, however, incorporated prespecified assessments of mitochondrial function or mitochondrial biomarkers; consequently, whether mitochondrial remodeling directly contributes to these clinical benefits remains unresolved. Reduction in major adverse cardiovascular events and histological improvement in MASH are clinically meaningful disease outcomes; neither, on its own, demonstrates a slowed trajectory of biological aging as defined in Section 1.1.

A related, and mechanistically informative, cardiovascular signal concerns cardiac rhythm. A 2026 meta‐analysis of randomized trials found that, among individuals with overweight or obesity, GLP‐1 RAs and co‐agonists were associated with an 18% relative reduction in atrial fibrillation risk versus placebo (risk ratio 0.82, 95% CI 0.70–0.96), with no significant difference across mono‐, dual‐, and triple‐agonist subgroups and no significant effect modification by magnitude of weight loss or concomitant SGLT2‐inhibitor use (Karakasis et al. 2026). This finding sits naturally alongside this review's central question of whether clinically relevant effects can be separated from weight loss alone: the absence of a weight‐loss‐magnitude interaction is intriguing but, consistent with the framework in Section 3.1, does not by itself establish a mitochondrial or otherwise receptor‐proximal mechanism, since AMPK‐independent systemic effects (on atrial inflammation, autonomic tone, or hemodynamics) remain plausible alternative explanations that have not been excluded.

### 4.3 Skeletal Muscle and the Lean Mass Controversy

Concerns about GLP‐1 RA‐induced muscle loss require careful phenotyping, because “lean mass,” “appendicular lean mass,” “skeletal muscle mass,” “muscle quality,” “strength,” and “physical function” are related but non‐interchangeable constructs that have often been conflated in this literature. DXA‐derived lean mass includes water, glycogen, connective tissue, and visceral organs in addition to contractile muscle, so a reduction in DXA lean mass does not necessarily equate to a proportional loss of contractile tissue, and this measurement limitation should not conversely be used to dismiss the potential clinical importance of sarcopenia, frailty, nutritional insufficiency, and functional decline in older adults treated with these agents.

A 2025 systematic review identified eight eligible preclinical studies of GLP‐1 RA effects on skeletal muscle mitochondrial function but no eligible human studies with direct mitochondrial endpoints (Old et al. 2025), a gap that persists despite continued preclinical interest. A network meta‐analysis of 22 randomized trials found that GLP‐1 RAs and GLP‐1/GIP co‐agonists significantly reduced total body weight, fat mass, and lean mass, with lean‐mass loss comprising approximately 25% of total weight loss and the percentage of lean mass relative to total body weight essentially unaffected; tirzepatide 15 mg and semaglutide 2.4 mg produced the greatest absolute fat loss but were least effective at preserving lean mass among the regimens studied (Karakasis et al. 2025). In a DXA substudy of SURMOUNT‐1, roughly one quarter of the weight lost with tirzepatide was lean mass (Look et al. 2025), raising concerns regarding muscle preservation.

Two recently published studies help contextualize these losses. A combined preclinical and human proof‐of‐concept study reported that in obese mice, GLP‐1 medicines reduced fat and liver mass more rapidly than skeletal muscle; while absolute muscle mass and strength decreased modestly, relative muscle mass and strength improved and running performance was maintained or improved, and the muscle proteome response to GLP‐1 medicines differed from that of calorie restriction despite similar total weight loss, arguing against a simple caloric‐restriction‐equivalence model of GLP‐1‐associated muscle change (Langer et al. 2026). Separately, an in vitro comparison of semaglutide, tirzepatide, and cagrilintide in C2C12 skeletal‐muscle myotubes under healthy and lipotoxic (palmitic‐acid‐treated) conditions is beginning to characterize agent‐specific mitochondrial responses at the cellular level, though these findings await confirmation in human muscle (Old et al. 2026).

**FIGURE 3:**
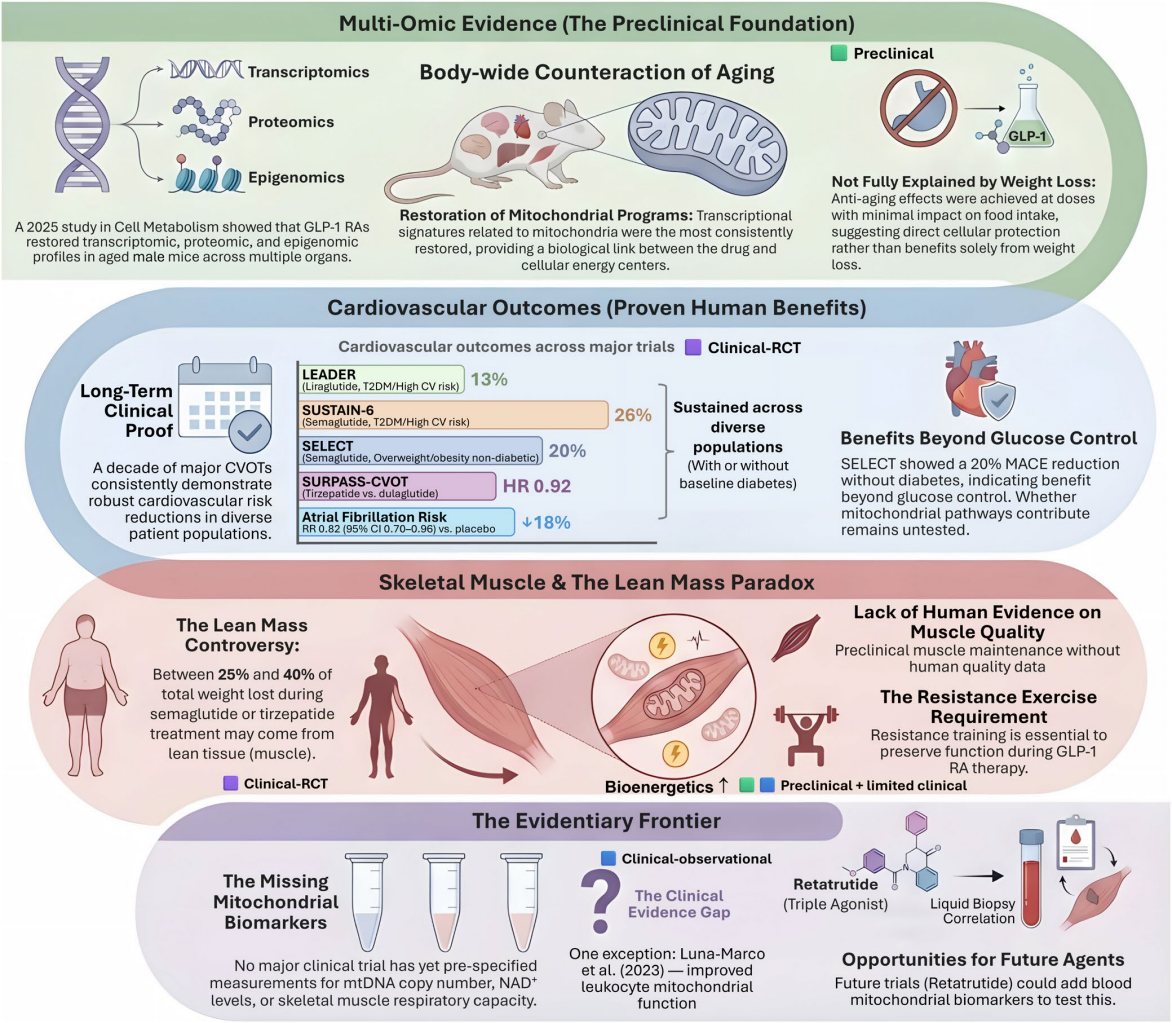
Clinical evidence supporting GLP‐1 RA‐mediated cardiovascular benefits and the mitochondrial evidence gap. A 2025 multi‐omic study demonstrated restoration of age‐associated molecular signatures in aged male mice (preclinical evidence), with mitochondrial pathways among the most consistently affected; this effect was not fully explained by the modest changes in food intake and body weight observed, though a contribution from these factors cannot be excluded. Major cardiovascular outcome trials—LEADER, SUSTAIN‐6, SELECT, and SURPASS‐CVOT (randomized controlled trials spanning a decade, 2016–2025)—demonstrated significant reductions in major adverse cardiovascular events (MACE) versus placebo or active comparator, and a 2026 meta‐analysis of randomized trials (clinical meta‐analysis) found an 18% relative reduction in atrial fibrillation risk with GLP‐1 receptor agonists and co‐agonists; collectively, these findings support clinical benefits beyond glucose lowering that the trials themselves were not designed to mechanistically attribute to mitochondrial remodeling. In skeletal muscle, preclinical and human proof‐of‐concept data (preclinical plus limited clinical evidence) indicate that relative muscle mass and function are maintained despite modest absolute reductions in lean mass; direct human data on muscle mitochondrial quality and respiratory capacity remain absent. No major clinical trial has incorporated prespecified mitochondrial biomarkers or functional endpoints (a current evidence gap; see Table 3 for proposed biomarker‐integrated trial designs), limiting assessment of whether mitochondrial remodeling directly contributes to clinical benefit; a small observational human dataset on leukocyte mitochondrial function (Luna‐Marco et al. 2023) is, to our knowledge, the most direct human mechanistic evidence currently available (Section 4.4). Evidence summarized in this figure spans preclinical, clinical‐observational, and randomized‐trial data of differing strength and receptor dependence; see Table 2 for the full evidence‐tier classification underlying each panel.

These data temper, but do not resolve, the muscle‐preservation question. They argue against the strongest form of the narrative that GLP‐1 RAs cause disproportionate muscle loss, but they do not establish that muscle quality or respiratory capacity per unit mass is preserved or improved in humans; this is not yet supported by direct human mitochondrial or respirometric data (Figure 3). If anything, the absence of direct human muscle‐quality data argues for exactly the kind of functional‐outcome trials proposed in Table 3 (Priority 3), particularly given evidence that resistance exercise combined with adequate protein intake can help preserve lean mass during GLP‐1 RA‐induced weight loss (Section 5.3). Whether next‐generation dual and triple agonists such as tirzepatide and retatrutide (Jastreboff et al. 2022; Jastreboff et al. 2023) alter this balance, and whether their hepatic benefits extend to MASLD (Sanyal et al. 2024), will require dedicated study. Clinical trials incorporating mitochondrial, functional, and physical performance endpoints are needed.

### 4.4 The Missing Biomarker: A Critical Evidence Gap

Despite extensive mechanistic evidence, no major cardiovascular or obesity outcome trial has incorporated prespecified assessments of mitochondrial function. Biomarkers such as mitochondrial DNA copy number (mtDNA‐CN), circulating NAD+ metabolites, and measures of mitochondrial respiratory capacity remain largely absent from clinical trial design. This absence of mitochondrial endpoints in the large outcome trials should be distinguished from the broader question of whether human mitochondrial evidence exists at all: it does, though it is small in scale and observational. In an ex vivo, treated‐versus‐untreated cohort of patients with T2DM, GLP‐1 RA treatment was associated with an improved leukocyte redox state, restored mitochondrial membrane potential and oxygen consumption, and reduced leukocyte‐endothelial interactions and carotid intima‐media thickness, a surrogate marker of atherosclerosis (Luna‐Marco et al. 2023). As an observational, non‐randomized comparison, this study cannot exclude confounding by indication or by the metabolic improvements that accompanied treatment, but it is, to our knowledge, among the most direct human mitochondrial‐function data currently available for GLP‐1 RAs, and we did not want the near‐total absence of mitochondrial endpoints in the large CVOTs to obscure it. Incorporating prespecified mitochondrial endpoints into future studies of GLP‐1‐based therapies could provide the first population‐scale evidence linking mitochondrial remodeling to clinical benefit. Establishing standardized mitochondrial biomarkers should therefore be a priority for future geroscience trials. The current state of clinical evidence supporting GLP‐1 RA‐mediated mitochondrial benefits is summarized in Figure 3 (Box 3).

BOX 3 **Key evidence gap.**

No published clinical trial of a GLP‐1 RA has incorporated a prespecified mitochondrial functional endpoint, such as mitochondrial DNA copy number (mtDNA‐CN), circulating NAD+ metabolites, or skeletal muscle respiratory capacity. This statement concerns prespecified endpoints in trial design specifically; it does not imply that no human mitochondrial data exist at all—a small observational human dataset is discussed in Section 4.4. As a result, whether mitochondrial remodeling directly contributes to the clinical benefits of GLP‐1‐based therapies remains unresolved. Incorporating standardized mitochondrial biomarkers alongside established cardiovascular, metabolic, and body‐composition outcomes could provide critical mechanistic insight and should be prioritized in the next generation of geroscience‐informed clinical trials.

## 5 Population Health Implications and Policy

The mechanistic and clinical evidence reviewed above suggests that GLP‐1 RAs may influence biological processes that extend beyond the treatment of individual cardiometabolic diseases. By targeting pathways linked to mitochondrial function, metabolic resilience, and aging, these therapies have the potential to affect health outcomes across multiple organ systems. However, translating these findings into population‐level benefit will require addressing several unresolved challenges, including equitable access, affordability, appropriate clinical indications, and integration with established lifestyle interventions. These issues will ultimately determine the public‐health impact of GLP‐1‐based therapies in aging populations.

### 5.1 Repositioning GLP‐1 RAs Within a Geroscience Framework

Geroscience proposes that targeting fundamental biological mechanisms of aging may yield broader health benefits than treating individual age‐related diseases in isolation (Sierra and Kohanski 2017). By engaging pathways linked to mitochondrial function, metabolic resilience, and cellular stress responses, GLP‐1 RAs have emerged as potential candidates for this approach. Whether they meet the geroscience‐intervention standard set out in Section 1.1, however, is a separate and currently unanswered question from whether they treat individual age‐related diseases effectively. Clinical evidence, including the SELECT trial, demonstrates cardiovascular benefit in individuals without diabetes, while preclinical studies suggest effects on molecular pathways associated with aging. Together, these findings raise the possibility that GLP‐1 RAs may have applications beyond conventional disease‐specific indications. Whether such benefits extend to broader aging populations remains unknown and will require prospective evaluation. Future studies may benefit from stratifying participants according to composite metabolic and mitochondrial risk profiles rather than established disease categories alone. As discussed in Section 4.1, enthusiasm must be tempered by negative clinical results: the EVOKE and EVOKE+ Phase 3 trials of oral semaglutide in early‐stage Alzheimer's disease did not meet their primary endpoint, with biomarker improvements failing to translate into slowed clinical progression (Cummings et al. 2026)—underscoring the gap between mechanistic promise and clinical outcomes, and illustrating precisely the distinction between disease treatment and aging modification developed in Section 1.1. If validated, this framework could support a shift toward earlier intervention aimed at preserving metabolic resilience and reducing the long‐term burden of age‐related chronic disease.

### 5.2 The Access and Equity Imperative

The potential population‐health impact of GLP‐1 RAs will depend not only on efficacy but also on equitable access. Current treatment costs remain prohibitive for many healthcare systems, particularly in low‐ and middle‐income countries (LMICs), which bear a growing share of the global burden of obesity, diabetes, and metabolic disease (Levi et al. 2026). Even in high‐income settings, uptake remains concentrated among wealthier and better‐insured populations despite a substantially larger eligible population (Kim et al. 2025). As patents expire and generic formulations emerge, access may expand substantially. Manufacturing‐cost analyses suggest that semaglutide could be produced at a fraction of current market prices (Levi et al. 2026). Policy approaches including tiered pricing, voluntary licensing, reimbursement expansion, and potential inclusion in the WHO Essential Medicines List may therefore warrant consideration (Pottie et al. 2026). Ensuring equitable availability will be critical if the benefits of GLP‐1–based therapies are to be realized at population scale rather than concentrated among already advantaged populations.

### 5.3 Synergy With Physical Activity

The AMPK‐SIRT1‐PGC‐1α pathway activated by GLP‐1 RAs overlaps substantially with the molecular signaling cascade induced by aerobic exercise. A randomized trial comparing exercise alone, liraglutide alone, and combination therapy demonstrated greater long‐term weight loss and metabolic improvement with the combined intervention than with either strategy alone, while exercise helped preserve lean mass (Lundgren et al. 2021). These findings suggest that GLP‐1‐based therapies may be most effective when integrated with structured physical activity rather than implemented as stand‐alone interventions. Defining the optimal combination of pharmacological and lifestyle approaches should be a priority for future healthy aging and geroscience research.

### 5.4 Mitochondrial Biomarkers for Population Studies

Blood‐based biomarkers, including mtDNA‐CN, circulating NAD^+^ metabolites, and cell‐free mitochondrial DNA, have been proposed as scalable measures of metabolic resilience and treatment response (Kennedy et al. 2014), though, as detailed in Section 2.3 and Table 1, each measure a distinct biological process and none is currently a validated surrogate for tissue mitochondrial function. Meta‐analyses indicate the association between mtDNA‐CN and cardiovascular outcomes is real but modest and heterogeneous (Fu et al. 2025; Li et al. 2024), and clinical trials of NAD+ precursors have so far produced inconsistent functional benefits (Prokopidis et al. 2025). Further validation is needed before routine clinical use. Ethical considerations surrounding privacy, consent, and data use should accompany their development.

## 6 Controversies, Gaps, and Future Directions

Although growing evidence supports a role for the GLP‐1–mitochondria axis in metabolic regulation and aging, major uncertainties remain. Five key controversies and five research priorities will determine whether the mechanistic promise of GLP‐1 RAs can be translated into clinically meaningful and equitable population‐health benefits.

### 6.1 Direct Versus Indirect Mitochondrial Effects

A central unresolved question is whether mitochondrial remodeling represents a direct consequence of GLP‐1 receptor signaling or a secondary adaptation to improved metabolic health (Box 2; see also the tiered framework introduced in Section 3.1 and applied in Table 2). Although cardiovascular protection is observed across categories of achieved weight loss in the SELECT trial (Lingvay et al. 2024), reductions in adiposity, lipotoxicity, and systemic inflammation may themselves improve mitochondrial function and contribute to clinical benefit. Determining the relative contribution of direct and indirect mechanisms is particularly important when considering applications in non‐obese or non‐diabetic aging populations. Resolving this question will require tissue‐specific GLP‐1R knockout models, longitudinal multi‐omic profiling, and clinical trials incorporating standardized mitochondrial endpoints.

### 6.2 Mitochondrial Quality Versus Muscle Mass

Whether improvements in mitochondrial quality compensate for reductions in lean mass during GLP‐1 receptor agonist treatment remains unresolved (Cruz‐Jentoft et al. 2019). This question is particularly relevant in older adults, for whom preservation of physical function may be more important than weight reduction alone. Current evidence is insufficient to determine whether gains in mitochondrial efficiency outweigh losses in muscle quantity. Resolving this controversy will require trials in adults aged 60 years and older incorporating functional outcomes, such as grip strength, gait speed, and cardiorespiratory fitness, alongside standardized mitochondrial endpoints.

### 6.3 Biomarker Readiness for Clinical Translation

Mitochondrial biomarkers are increasingly proposed as tools for risk stratification and treatment monitoring, yet their clinical utility remains incompletely established. Measures such as mitochondrial DNA copy number (mtDNA‐CN), circulating NAD^+^ metabolites, and cell‐free mitochondrial DNA require further standardization, validation, and evaluation across diverse populations. Whether these biomarkers can serve as reliable surrogate endpoints for mitochondrial health and treatment response remains an important translational question (Table 1).

### 6.4 Applicability to Non‐Obese Aging Populations

GLP‐1 RAs are currently approved for obesity and T2DM. The risk–benefit profile in nonobese, nondiabetic older adults may differ substantially from that in metabolically compromised individuals, particularly given concerns regarding lean mass loss, bone health, and gastrointestinal adverse effects (Pantazopoulos et al. 2025)—discussed further, together with additional risks specific to older populations, in new Section 6.5. The relative mitochondrial effects of dual and triple agonists (tirzepatide and retatrutide) versus mono‐agonists are also unknown and merit head‐to‐head evaluation (Jastreboff et al. 2022; Sanyal et al. 2024). Unlike GLP‐1R, GIPR mRNA is widely expressed in ventricular and atrial myocardium in both rodents and humans, supporting a plausible direct cardiac role for GIP signaling that has not been mechanistically characterized in the mitochondrial context discussed in this review; whether GIP‐receptor engagement by dual and triple agonists contributes independently to the mitochondrial and body‐composition effects described in Sections 3, 4, or whether these effects are attributable chiefly to the shared GLP‐1‐receptor component, is an open question requiring receptor‐specific pharmacological or genetic dissection. Addressing these questions will require dedicated clinical trials, appropriate safety‐monitoring frameworks, and careful evaluation of clinically meaningful aging outcomes (Table 3).

### 6.5 Risk Considerations Specific to Older and Non‐Obese Populations

The risk–benefit calculus for a hypothetical geroscience indication in nonobese, non‐diabetic older adults differs materially from that established in the severely obese populations studied to date. Relevant risks include gastrointestinal intolerance (nausea, vomiting, and delayed gastric emptying, which may be more consequential in frail individuals with reduced physiological reserve); dehydration, particularly during dose escalation or intercurrent illness; inadequate protein and micronutrient intake accompanying reduced caloric intake, which could compound rather than mitigate sarcopenia risk in already‐frail individuals; a rate of lean‐mass loss that, while proportionate to weight loss (Section 4.3), occurs from a lower baseline muscle reserve in older adults; gallbladder disease; the pharmacoeconomic and adherence burden of long‐term injectable therapy, including weight regain and reversal of metabolic gains on discontinuation; possible bone‐health effects that remain incompletely characterized; and the potential for adverse drug–drug interactions in a population with high rates of polypharmacy. These considerations argue against extrapolating the risk–benefit profile established in obesity and T2DM populations to a hypothetical future indication in nonobese older adults, and underscore the need for dedicated safety studies in this population before any geroscience indication could be responsibly considered (Table 3, Priority 3).

## 7 Conclusions

Mitochondrial dysfunction is increasingly recognized as a central feature of metabolic aging, linking impaired bioenergetics, disrupted quality control, chronic inflammation, and loss of metabolic resilience. As reviewed here, accumulating evidence suggests that GLP‐1 RAs engage these pathways through a proposed GLP‐1–mitochondria axis involving AMPK, PGC‐1α, and NAD+‐dependent signaling in specific, tissue‐ and stressor‐dependent contexts (Table 2). By influencing mitochondrial biogenesis, dynamics, and mitophagy where these effects have been directly demonstrated, GLP‐1 RAs may affect biological processes that extend beyond glycemic control and weight management. Current clinical and preclinical evidence provides a rationale for investigating, rather than confirming, GLP‐1 RAs within a geroscience framework as defined in Section 1.1. Cardiovascular benefits in individuals without diabetes, broad multi‐omic age‐associated molecular changes in male animal models, and convergent mechanistic studies collectively support the possibility that mitochondrial remodeling contributes to the therapeutic effects of these agents. However, definitive evidence remains lacking. No major clinical trial has incorporated prespecified mitochondrial endpoints, and the relative contributions of direct receptor signaling, weight loss, and broader metabolic improvements remain unresolved and, in most tissues, untested against receptor antagonism or genetic deletion.

Future progress will depend on integrating standardized mitochondrial biomarkers into clinical trials, clarifying causal mechanisms across tissues and populations, and ensuring that advances in GLP‐1‐based therapies are implemented equitably and in combination with established lifestyle interventions. Addressing these challenges will determine whether the GLP‐1–mitochondria axis can be translated into effective, scalable, and evidence‐based strategies for promoting healthy aging and reducing the population burden of metabolic disease.

## Author Contributions

R.C., A.P.T., and C.‐J.L. conceived the overall concept of the study, while A.P.T. and B.W. contributed to the development of the original idea. R.C. and C.‐J.L. prepared the manuscript draft; R.C., A.P.T., and C.‐J.L. participated in the review and revision of the manuscript. All authors discussed the results and contributed to the final version of the manuscript.

## Funding

This work was supported by the National Science and Technology Council, 114‐2628‐B075‐B‐001‐MY3; Kaohsiung Veterans General Hospital, KSVGH‐114‐056, 148.

## Conflicts of Interest

The authors declare no conflicts of interest.

## Data Availability

Data sharing not applicable to this article as no datasets were generated or analysed during the current study.
